# Trends and hotspots in pulmonary fibrosis biomarker research: a bibliometric analysis

**DOI:** 10.3389/fmed.2025.1541364

**Published:** 2025-04-25

**Authors:** Ling Wang, Jianliang Huang, Gan Tan, Yurou Zhang, Mingkai Xia, Zhen Kang, Yun Xiao, Mingsheng Lei

**Affiliations:** ^1^Department of Pulmonary and Critical Care Medicine, Zhangjiajie Hospital Affiliated to Hunan Normal University, Zhangjiajie, Hunan, China; ^2^Biomedical Research Institute, Zhangjiajie College, Zhangjiajie, Hunan, China; ^3^The Second Affiliated Hospital of Hunan University of Chinese Medicine, Changsha, Hunan, China; ^4^Department of Pulmonary and Critical Care Medicine, Changsha Central Hospital, Changsha, Hunan, China

**Keywords:** pulmonary fibrosis, biomarkers, disease management, bibliometric analysis, MUC5B

## Abstract

**Background:**

Pulmonary fibrosis is a fatal disease characterized by progressive scarring of lung tissue, with a complex pathogenesis and limited therapeutic options. The identification of robust biomarkers is critical for addressing key clinical challenges, including delayed diagnosis and poor prognostic assessment.

**Methods:**

This study systematically analyzes global research trends and emerging hotspots in pulmonary fibrosis biomarkers by examining literature from 2001 to 2024 indexed in the Web of Science Core Collection. Utilizing a suite of bibliometric tools including VOSviewer, CiteSpace, Bibliometrix, Scimago Graphica, and OriginPro 2021, this work provides the first comprehensive insight into the evolving landscape of biomarker research in pulmonary fibrosis.

**Results:**

This study included a total of 2,519 articles and reviews related to pulmonary fibrosis biomarkers. Since 2005, publication trends in this field have steadily increased. Research on pulmonary fibrosis biomarkers has involved 71 countries, 3,036 institutions, 760 journals, and over 14,000 researchers. China produced the highest number of publications (*n* = 535, 21.2%, TLCS = 459), followed by the United States (*n* = 529, 21%, TLCS = 3,527) and Japan (*n* = 270, 10.7%, TLCS = 1,279), with the United States exerting the greatest influence. The UNIVERSITY OF CALIFORNIA SYSTEM (*n* = 164) and HARVARD UNIVERSITY (*n* = 141) contributed the largest bodies of work. The most prolific authors in this domain are BARGAGLI E (*n* = 45), MAHER TM (*n* = 42), and MARTINEZ FJ (*n* = 32). The AMERICAN JOURNAL OF RESPIRATORY AND CRITICAL CARE MEDICINE is widely regarded as the leading journal in this field. In recent years, research has increasingly focused on macrophages, computed tomography, and Muc5b promoter polymorphism, among other areas. The concept of “double blind” reflects the translational trend of biomarkers toward clinical applications, particularly their potential utility in acute exacerbations of pulmonary fibrosis, interstitial pulmonary fibrosis, cystic pulmonary fibrosis, and radiation-induced pulmonary fibrosis.

**Conclusion:**

The clinical application of gene and imaging biomarkers achieved through the integration of multiple parameters and multi-omics fusion represents a promising future trend and emerging hotspot in pulmonary fibrosis biomarker research.

## Introduction

Pulmonary fibrosis is an interstitial lung disease characterized by fibroblast proliferation, extracellular matrix deposition, and decreased lung compliance. According to recent statistics, its global incidence ranges from 1 to 31.5 per 100,000 individuals annually, with a prevalence of 6.3 to 71 per 100,000. Ranked as the 40th leading cause of death worldwide, the disease confers a median post-diagnosis survival of only 2–6 years (1–3), severely impairing patient quality of life and imposing a substantial economic burden. Clinically, managing pulmonary fibrosis presents significant challenges. The underlying pathogenesis remains only partially elucidated, with most cases having an unclear etiology that likely involves a complex interplay of genetic factors, environmental exposures, aging, and aberrant repair processes. This uncertainty complicates the identification of definitive therapeutic targets (4). Moreover, the disease exhibits marked heterogeneity, with distinct subtypes demonstrating varied pathological mechanisms; although personalized treatment is theoretically optimal, the absence of refined classification criteria hinders precise intervention. Therapeutically, no curative options currently exist. Approved antifibrotic agents such as nintedanib and pirfenidone merely decelerate disease progression without reversing fibrosis. Their high cost, coupled with suboptimal patient responses and significant side effects (e.g., nintedanib-associated diarrhea and hepatotoxicity, pirfenidone-induced photosensitivity and gastrointestinal disturbances) (5, 6), underscores the urgent need for novel therapeutic targets and treatments.

The unpredictability of disease progression poses a significant challenge to clinical management, compounded by the absence of reliable predictive tools. Currently, the diagnosis and monitoring of pulmonary fibrosis predominantly rely on imaging techniques (e.g., HRCT) and pulmonary function tests (such as FVC and DLCO). However, the lack of molecular biomarkers for early diagnosis and prognostic assessment hinders timely intervention. Biomarkers, as critical indicators that reflect the underlying pathophysiological alterations at the molecular level, have garnered increasing attention in pulmonary fibrosis management. For instance, studies have shown that elevated protein levels in bronchoalveolar lavage fluid, which correlate positively with lymphocyte counts, can assist in the diagnosis and classification of the disease (7, 8). Moreover, TGF-*β* plays a crucial role in the pathogenesis of pulmonary fibrosis by promoting fibroblast proliferation and collagen synthesis, thereby driving disease progression (9). Clinically, TGF-β not only serves as an indicator for evaluating therapeutic efficacy but also aids in assessing disease severity and progression. Similarly, genetic variants in TOLLIP and MUC-5B are closely associated with disease susceptibility, clinical manifestations, and prognosis; monitoring these variants can facilitate risk stratification and personalized treatment approaches (10). Furthermore, the convergence of multi-omics technologies is fostering the development of a multidimensional biomarker framework encompassing molecular, histological, imaging, and functional parameters that holds promise for enhancing precision in early screening, target identification, dynamic monitoring, and prognostic evaluation of pulmonary fibrosis.

This study employs bibliometric and visualization approaches to thoroughly assess the research landscape of pulmonary fibrosis biomarkers from 2001 to 2024. The bibliometric analysis utilizes software tools such as CiteSpace, VOSviewer, and HistCite to evaluate the published literature quantitatively. It visually represents research trends, emerging hotspots, and potential future directions (11, 12). To date, no study has utilized bibliometric methods to comprehensively analyze the progression and trends in pulmonary fibrosis biomarkers.

## Methods

### Data collection

**Figure 1:**
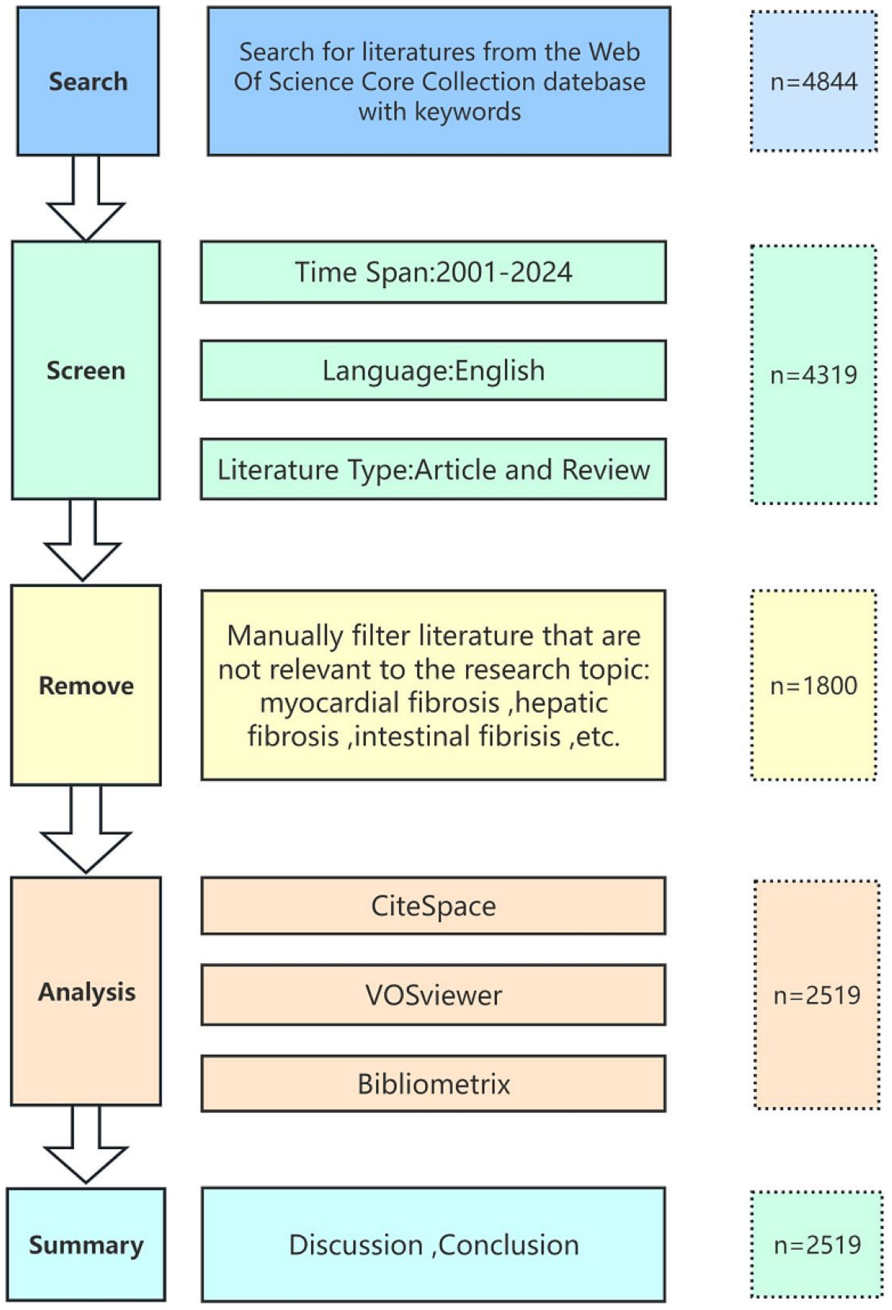
Flowchart of data processing.

We retrieved relevant literature on pulmonary fibrosis biomarkers from the Web of Science Core Collection (WOSCC) spanning from January 2001 to August 2024. To ensure data accuracy, all searches were completed on August 20, 2024, as illustrated in Figure 1. The search query was formulated as follows: TS = (Pulmonary Fibrosis OR Pulmonary Fibroses OR Fibrosing Alveolitides OR Fibrosing Alveolitis OR Idiopathic Diffuse Interstitial Pulmonary Fibrosis) AND TS = (Biomarkers OR Biomarker OR Biological Marker OR Biological Markers OR Biologic Markers OR Biologic Marker OR Clinical Marker OR Clinical Markers OR Surrogate Markers OR Surrogate Marker OR Surrogate Endpoints OR Surrogate End Points OR Surrogate Endpoint OR Surrogate End Point OR Immune Marker OR Immune Markers OR Immunologic Marker OR Immunologic Markers OR Laboratory Marker OR Laboratory Markers OR Serum Markers OR Serum Marker OR Viral Markers OR Viral Marker OR Biochemical Marker OR Biochemical Markers). We limited our search to English-language articles and reviews, thereby excluding conference abstracts, editorials, and proceedings. Each record was manually screened to remove irrelevant publications. Ultimately, the selected records including full bibliographic details and cited references (e.g., authors, countries, institutions, references, keywords, and journals) were exported in Plain Text format for subsequent bibliometric and visualization analyses.

### Data analysis

This study employs several software tools, including VOSviewer (version 1.6.20), CiteSpace (version 6.3), the R package “Bibliometrix” (version 4.1.2), Scimago Graphica, and OriginPro 2021, for data analysis and visualization of the included literature. VOSviewer is a specialized tool for constructing and visualizing bibliometric networks. By analyzing citation data, it generates co-citation networks, collaboration networks, and citation networks, providing a clear visualization of the knowledge structure in a specific research field. In this study, VOSviewer (version 1.6.20) was used to analyze and visualize the countries, institutions, journals, authors, and keywords of the included literature. The Total Global Citation Score (TGCS) and Total Local Citation Score (TLCS) are key indicators for evaluating the impact of scientific research. TGCS measures the global citation frequency of a paper, reflecting its broad influence in the international academic community and its contribution to global scholarly research. TLCS, on the other hand, focuses on the citations within the specific research domain, quantifying the paper’s academic value and influence within its discipline or research area. CiteSpace is an advanced analytical tool specifically designed for bibliometrics and scientific knowledge mapping, widely used in academic research and trend prediction. It transforms large volumes of bibliometric data into graphical networks, offering an intuitive display of the knowledge structure, thematic associations, and evolutionary paths in a research field. Furthermore, CiteSpace reveals temporal trends in a specific domain, helping to identify emerging topics. Thus, in this study, CiteSpace (version 6.3) was employed to analyze and visualize co-cited journals, co-cited authors, research trends, and hotspots in the literature. The R package “Bibliometrix” supports the importation of literature data from multiple databases, allowing for the statistical analysis of publication volume and citation counts. It also facilitates trend analysis, enabling efficient identification of research hotspots and trends within a specific field. In this study, “Bibliometrix” (version 4.1.2) was used to perform statistical analysis of the included literature data, as well as to examine the evolution and coupling of related topics. OriginPro is a powerful data processing tool that supports multi-tool data interaction and can generate a variety of complex graphs. In this study, OriginPro 2021 was utilized for the visualization of publication volume analysis. Scimago Graphica is a tool focused on the visualization of scientific data. It supports the analysis of scientific literature, research performance, collaboration networks, and more. It can be used to identify research trends and emerging fields, and to generate scientific maps that depict the structure and evolution of research domains. Therefore, in this study, we combined VOSviewer (version 1.6.20) and Scimago Graphica to create time-zone maps of keywords, analyzing their evolutionary processes, identifying emerging keywords, and exploring the frontiers of biomarker research in pulmonary fibrosis.

## Results

### Global publication trends and country analysis

**Figure 2:**
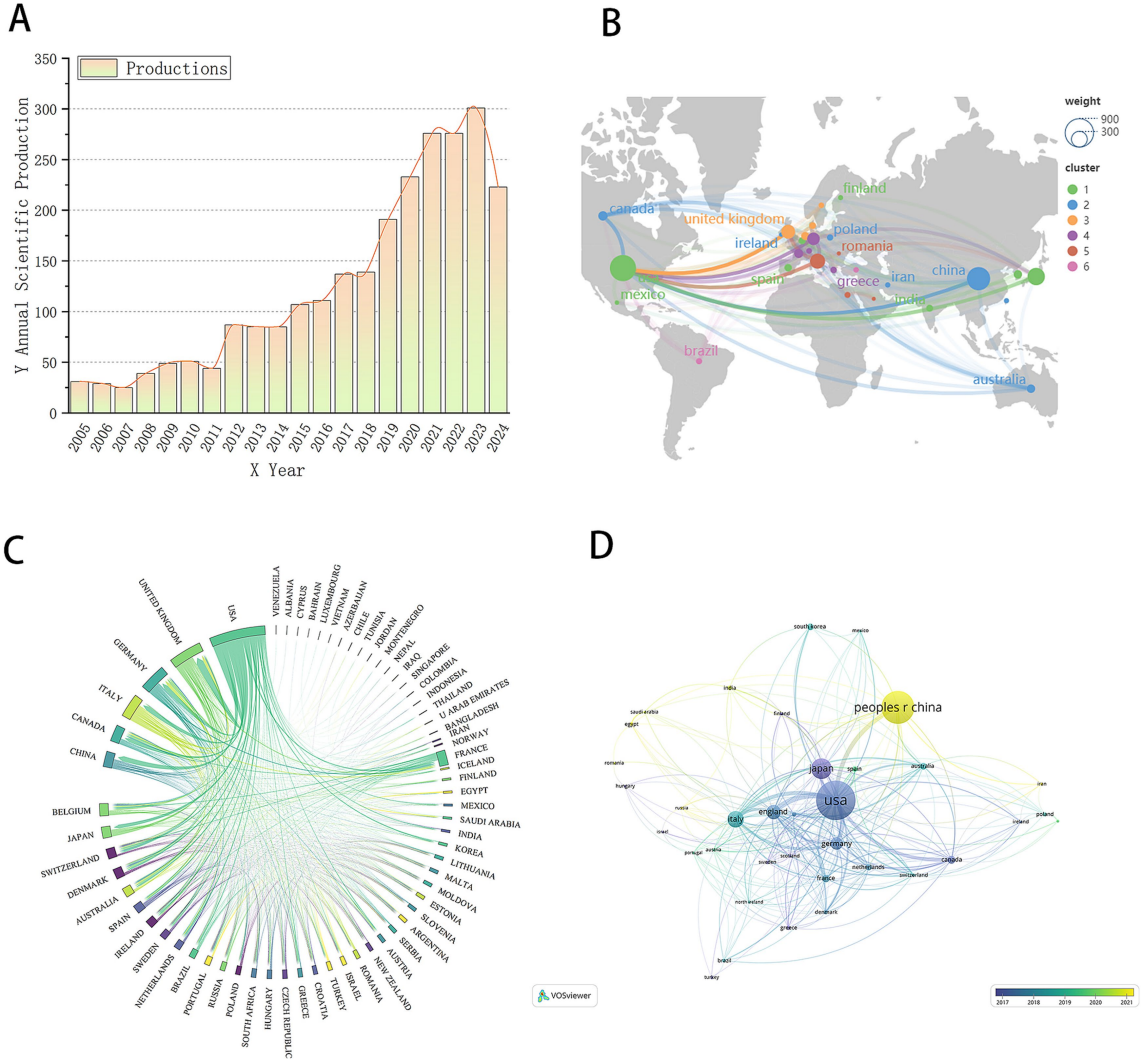
**(A)** Trend chart of the number of papers published by the year. **(B)** World geographic map of the country. **(C)** Country chord diagram. **(D)** National document cooperation time map.

This study includes 2,519 papers published between 2001 and 2024 on biomarkers of pulmonary fibrosis. Due to the timing of the search, the number of publications for 2024 is incomplete. We employed Bibliometrix to conduct data statistics on the included literature and extracted publication volume data, which was then visualized using Origin software (Figure 2A). As shown in Figure 2A, with the exception of 2007 and 2011, where there was no significant increase in publications, the number of publications for each subsequent year has generally increased compared to the previous year. The overall trend indicates a steady rise in publication volume, suggesting a growing focus on pulmonary fibrosis biomarkers. This annual increase in publication output indicates that global research on pulmonary fibrosis biomarkers is becoming progressively deeper. The field is currently in a phase of rapid development, with sustained research enthusiasm, reflecting the academic community’s growing attention to biomarkers in pulmonary fibrosis. This trend also suggests the potential for future groundbreaking advancements in the field, providing stronger support for the diagnosis, treatment, and prognosis of pulmonary fibrosis.

National analysis revealed that a total of 71 countries have contributed to research on biomarkers of pulmonary fibrosis. We compiled the publication volume data by country/region, as shown in Table 1. The country with the highest publication volume is China (*n* = 535, 21.2%, TLCS = 459), followed by the United States (*n* = 529, 21%, TLCS = 3,527), Japan (*n* = 270, 10.7%, TLCS = 1,279), and Italy (*n* = 173, 6.9%, TLCS = 757). According to the data, China has the highest number of publications, yet its influence appears relatively modest. In contrast, although the United States does not have the highest publication volume, it has the most citations, indicating a deeper engagement with research in this field. This highlights the significant role of the United States in pulmonary fibrosis biomarker research and suggests that, while China leads in publication volume, there is room for improvement in the innovation and quality of Chinese research output.

**Table 1:** Top 10 countries/institutions by publication volume.

Number	Country	Papers	Proportion%	TLCS	Number	Institution	Papers
1	CHINA	535	21.2	459	1	UNIVERSITY OF CALIFORNIA SYSTEM	164
2	USA	529	21	3,527	2	HARVARD UNIVERSITY	141
3	JAPAN	270	10.7	1,279	3	UNIVERSITY OF MICHIGAN	128
4	ITALY	173	6.9	757	4	UNIVERSITY OF MICHIGAN SYSTEM	128
5	UNITED KINGDOM	117	4.6	900	5	EGYPTIAN KNOWLEDGE BANK (EKB)	122
6	GERMANY	85	3.4	701	6	UNIVERSITE PARIS CITE	119
7	FRANCE	64	2.5	298	7	PENNSYLVANIA COMMONWEALTH SYSTEM OF HIGHER EDUCATION (PCSHE)	116
8	KOREA	61	2.4	322	8	ASSISTANCE PUBLIQUE HOPITAUX PARIS (APHP)	109
9	CANADA	54	2.1	330	9	UNIVERSITY OF PITTSBURGH	104
10	NETHERLANDS	44	1.7	459	10	IMPERIAL COLLEGE LONDON	88

To gain a more intuitive understanding of the global distribution and geographical locations of the countries involved in pulmonary fibrosis biomarker research, we created a geographic map of the countries associated with the included literature (Figure 2B). From the map, it is evident that the majority of countries conducting research in this field are located in Europe and North America. This distribution pattern is likely linked to the resource investments, research infrastructure, and academic traditions prevalent in these regions. Subsequently, we created a chord diagram to visualize the collaboration between countries (Figure 2C). The chord diagram not only illustrates the collaborative relationships between countries but also indicates the strength of cooperation through the thickness of the lines. As shown in Figure 2C, the United States and the United Kingdom have the most robust collaboration, suggesting frequent academic exchanges and joint research projects between the two countries in the field of pulmonary fibrosis biomarkers. In addition, the United States maintains extensive collaboration with countries such as China and Canada. This broad network of international cooperation is a key factor contributing to the United States’ significant influence in the field. Through such collaborations, the United States is able to integrate global resources, driving further advancement in research. To further explore the timeline of research activities by country in the field of pulmonary fibrosis biomarkers, we selected the top 36 countries by publication volume and plotted a collaboration network timeline (Figure 2D). The timeline reveals that the United States, Japan, and Germany were among the earliest countries to engage in this research area, and these countries have maintained close collaboration with each other. In contrast, China began research in this field later, which may explain the relatively lower international impact of Chinese publications. Moreover, China’s international collaborations are somewhat limited and lack a broad network, which has, to some extent, hindered the flow of knowledge and technological advancements, thus affecting the global influence of its research output.

### Institutional and author analysis

**Figure 3:**
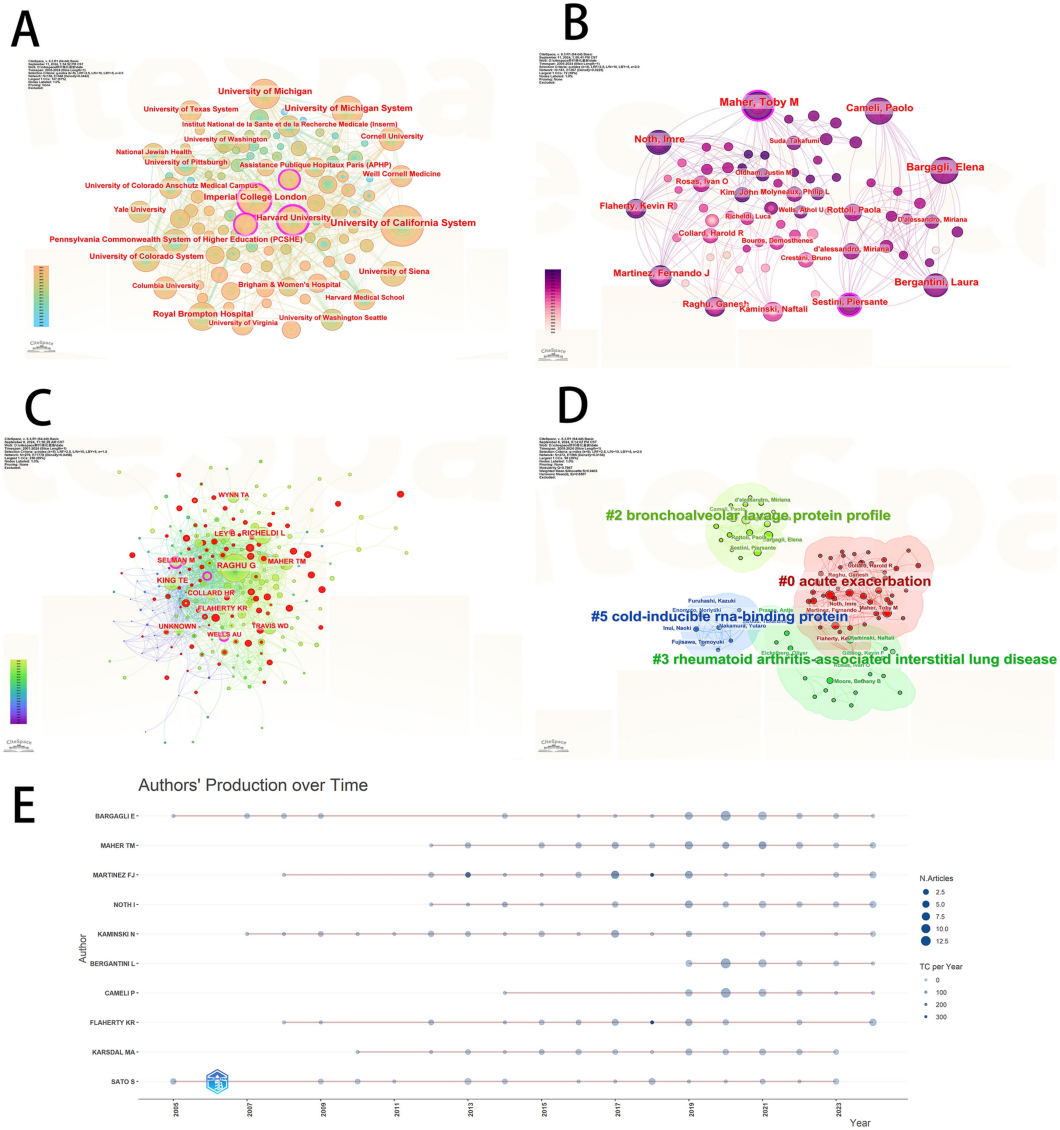
**(A)** Institutional collaboration diagram. **(B)** Author collaboration network diagram. **(C)** Authors co-cited analysis chart. **(D)** Authors’ cluster analysis plot. **(E)** Authors’ production over time map.

Research on biomarkers of pulmonary fibrosis is conducted across 3,036 institutions globally. As shown in Table 1, the institution with the highest number of publications is the University of California System (*n* = 164), followed by Harvard University (*n* = 141), highlighting the significant contribution of the University of California System to the field of pulmonary fibrosis biomarker research. To examine the collaboration relationships between institutions, we created an institutional collaboration network diagram (Figure 3A). The size of each node corresponds to the level of collaboration, with larger nodes indicating closer collaboration and greater institutional significance. As shown in Figure 3A, the University of California System, Harvard University, and Imperial College London are the three largest nodes, suggesting that these institutions have the most extensive collaboration with others, and that they also maintain broad cooperative relationships among themselves.

Over 14,000 individuals have contributed to research on biomarkers of pulmonary fibrosis. We compiled a list of the top 10 authors by publication volume, as shown in Table 2. The author with the highest number of publications is E. Bargagli (*n* = 45, TLCS = 302), followed by T.M. Maher (*n* = 42, TLCS = 444), and F.J. Martinez (*n* = 32, TLCS = 833). Among all authors, F.J. Martinez has the highest Local Citation Score (TLCS = 833). As shown in the table, F.J. Martinez not only ranks highly in terms of publication volume but also has the most citations, establishing him as an authoritative figure in the field of idiopathic pulmonary fibrosis (IPF) research. F.J. Martinez led the development of the “An Official ATS/ERS/ALAT Statement: Idiopathic Pulmonary Fibrosis: Evidence-based Guidelines for Diagnosis and Management,” which systematically summarizes current understanding of the disease’s characteristics and emphasizes the crucial role of imaging and biomarkers in its diagnosis and management. His team identified TGF-*β* as a key regulatory factor in the fibrotic process following lung injury (9), and biomarkers such as SP-D, MMP-7, and osteopontin significantly improve the diagnostic accuracy of IPF (13, 14). Although these biomarkers are not yet widely applied in clinical practice, their findings have laid an important theoretical and experimental foundation for establishing a biomarker-based diagnostic and therapeutic framework for pulmonary fibrosis, providing a scientific basis for the development of more precise diagnostic methods and personalized treatment options in the future.

**Table 2:** Total publications and citations of authors.

Number	Authors	Articles	Articles fractionalized	TLCS
1	BARGAGLI E	45	5.85	302
2	MAHER TM	42	5.70	444
3	MARTINEZ FJ	32	2.30	833
4	NOTH I	32	2.79	246
5	KAMINSKI N	31	4.01	525
6	BERGANTINI L	30	3.60	62
7	CAMELI P	29	3.43	87
8	FLAHERTY KR	29	2.12	675
9	KARSDAL MA	29	3.52	131
10	SATO S	29	2.98	105

To examine the collaboration relationships between authors, we created an author collaboration network diagram (Figure 3B). As shown in the diagram, T.M. Maher, E. Bargagli, and P. Cameli are the three largest nodes, indicating that these authors have the most extensive collaboration with others and highlighting their significant contributions to the field of pulmonary fibrosis biomarker research. Co-citation refers to instances where two or more authors are cited together in the same literature. A higher frequency of co-citation indicates a stronger thematic connection between authors. Analyzing highly co-cited authors can thus help identify key research themes and focal issues within a field. Using CiteSpace, we conducted a co-citation analysis of authors and generated an author co-citation network map (Figure 3C). In this network, the size of each node reflects the frequency of co-citation; the larger the node, the more frequently the author is co-cited. As shown in Figure 3C, the largest node belongs to G. Raghu, indicating that he is the most frequently co-cited author in this field. We further examined the literature authored by G. Raghu and identified several key contributions, particularly in his recent 2023 publication. In this work, G. Raghu highlights critical challenges in the clinical management of pulmonary fibrosis. First, he underscores the considerable individual variability in disease progression, which remains difficult to predict with precision. Second, while current pharmacological treatments can slow disease progression, they require ongoing assessment through regular physiological monitoring and serial imaging evaluations to adapt therapy dynamically (15). However, the lack of precise physiological parameters and universally applicable imaging features in clinical settings presents a major barrier. This gap contributes to the unpredictability and limited controllability of pulmonary fibrosis progression.

Cluster analysis is a method of grouping similar objects into distinct clusters based on static analysis. The size of a cluster reflects the number of samples it contains, while the density indicates the strength of association. By conducting cluster analysis of authors, we can quickly identify core researchers in various subfields, providing important guidance for selecting future research directions and potential collaboration partners. We generated an author clustering diagram (Figure 3D), which displays four primary clusters, each represented by a different color. As shown in Figure 3D, these clusters correspond to four major research directions: Acute exacerbation (red, #0), Bronchoalveolar lavage protein profile (light green, #2), Rheumatoid arthritis associated interstitial lung disease (CTD-ILDs; dark green, #3), and Cold-inducible RNA-binding protein (CIRBP; blue, #5).Acute exacerbation is a critical research direction in pulmonary fibrosis, often associated with poorer prognosis. Therefore, identifying biomarkers that can predict and assess the risk of acute exacerbation is essential for effective disease management. BALF proteomics represents another significant area of research in pulmonary fibrosis biomarkers. By analyzing the protein composition in BALF, researchers can gain deeper insights into the pathophysiological processes of pulmonary fibrosis. Studies have shown that various proteins in BALF, such as surfactant proteins A (SP-A) and D (SP-D), hold potential value in the diagnosis and risk prediction of pulmonary fibrosis (16). CTD-ILDs is an important branch of pulmonary fibrosis research. Currently, research on CTD-ILDs primarily focuses on identifying biomarkers that can distinguish different subtypes of CTD-ILDs, as well as evaluating indicators of disease activity and prognosis (17). Cold-inducible RNA-binding protein (CIRBP) is a relatively recent focus in pulmonary fibrosis research. CIRBP plays a crucial role in cellular stress responses, and its expression levels in pulmonary fibrosis correlate with disease severity, making it a potential biomarker for monitoring disease progression and assessing treatment efficacy (18).

A temporal analysis of the publication trajectories of the top 10 most prolific authors (Figure 3E) was conducted to systematically examine their research activity and output patterns within the field. The size of each node is positively correlated with the number of publications in a given year, while the length of the connecting lines reflects the duration of sustained research activity. Among the authors, E. Bargagli demonstrated the most extended research continuity, consistently contributing to the field over multiple years. In contrast, L. Bergantini exhibited a more recent entry into the domain, with publications beginning in 2019. This temporal distribution provides an objective basis for distinguishing established experts from emerging scholars, offering valuable insights into individual research lifecycles. Such information is instrumental in identifying potential collaborators and informing strategic decisions regarding future research directions.

### Journal analysis

**Figure 4:**
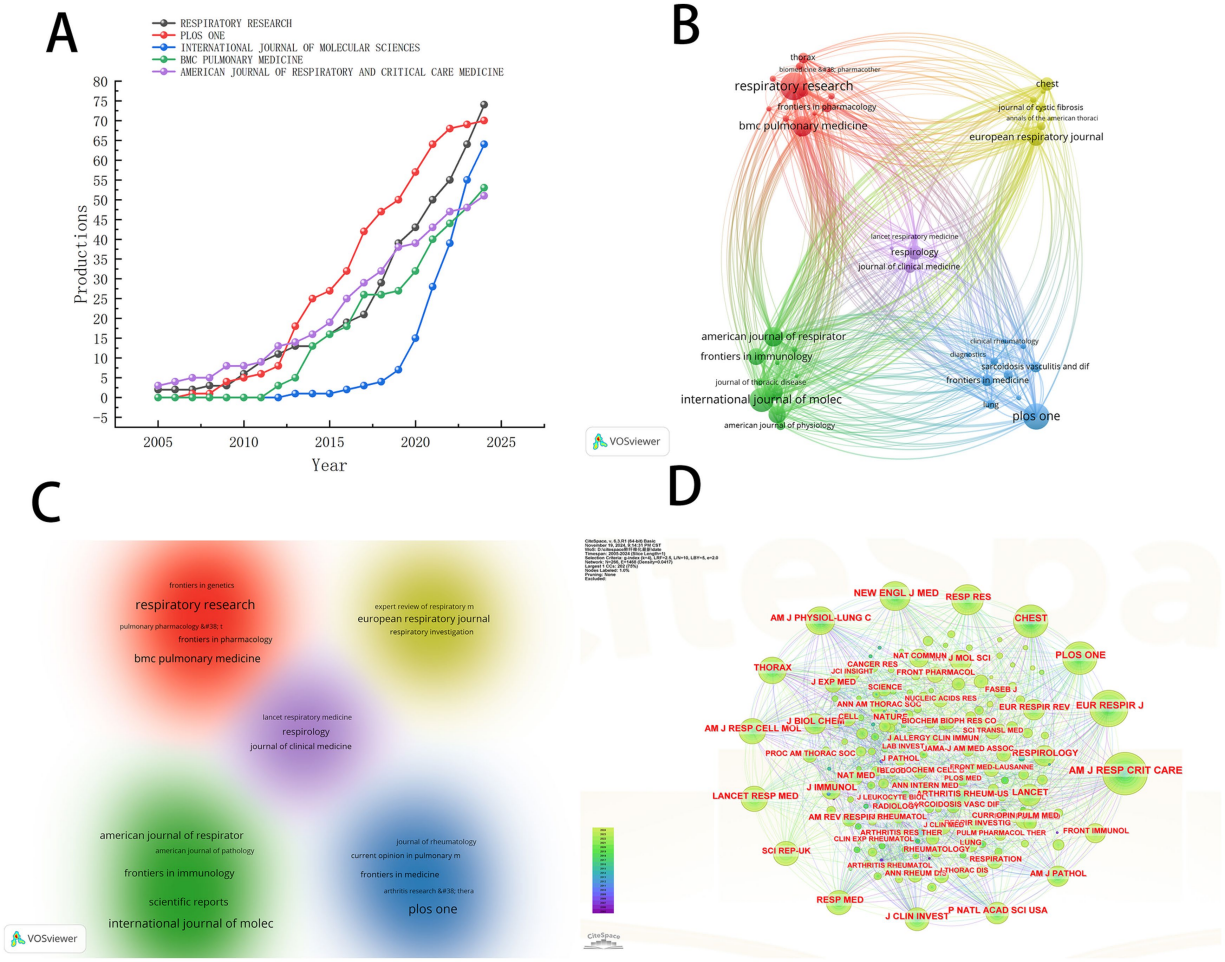
**(A)** The trend chart of the top five journals by the number of published papers. **(B)** Journal collaboration diagram. **(C)** Journal clustering diagram. **(D)** Journal co-citation analysis chart.

Research on pulmonary fibrosis biomarkers has been published in 760 different journals. Table 3 lists the top 10 journals by publication volume. Respiratory Research is the journal with the highest number of publications in this field (*n* = 74), followed by PLOS ONE (*n* = 70) and the International Journal of Molecular Sciences (*n* = 64). Notably, American Journal of Respiratory and Critical Care Medicine has the highest H-index (h_index = 35), suggesting that it may be regarded as an authoritative journal widely recognized within the field. We visualized the publication trends of the top five journals by publication volume (Figure 4A). As shown in Figure 4A, PLOS ONE was the leading journal in terms of publications after 2012, until it was surpassed by Respiratory Research in 2023. To explore collaboration patterns between journals, we constructed a journal collaboration network diagram (Figure 4B). The diagram reveals significant collaboration between Respiratory Research and BMC Pulmonary Medicine, while PLOS ONE has also formed a close partnership with Lung. Subsequently, we performed cluster analysis of the journals and generated a network relationship map (Figure 4C). Journals within the same cluster indicate a similar thematic focus on pulmonary fibrosis biomarkers, assisting in the identification of journals researching related topics. The results show that all journals were categorized into five distinct clusters, with Respiratory Research and BMC Pulmonary Medicine sharing similar research themes.

**Table 3:** Top 10 journals by publication volume.

Number	Sources	Articles	h_index
1	RESPIRATORY RESEARCH	74	29
2	PLOS ONE	70	29
3	INTERNATIONAL JOURNAL OF MOLECULAR SCIENCES	64	15
4	BMC PULMONARY MEDICINE	53	20
5	AMERICAN JOURNAL OF RESPIRATORY AND CRITICAL CARE MEDICINE	51	35
6	SCIENTIFIC REPORTS	48	15
7	EUROPEAN RESPIRATORY JOURNAL	45	29
8	RESPIRATORY MEDICINE	45	22
9	FRONTIERS IN IMMUNOLOGY	44	14
10	RESPIROLOGY	37	20

To identify the most significant journals, we conducted a co-citation analysis of the journals included in the literature and generated a network map (Figure 4D). The larger the node, the more frequent the co-citations. As shown in Figure 4D, the American Journal of Respiratory and Critical Care Medicine exhibits the highest co-citation frequency, further confirming its authoritative position in the field of pulmonary fibrosis biomarker research. This finding corroborates the results of the H-index analysis, collectively establishing this journal as the most important platform for knowledge dissemination in the study of pulmonary fibrosis biomarkers.

### Literature analysis

**Figure 5:**
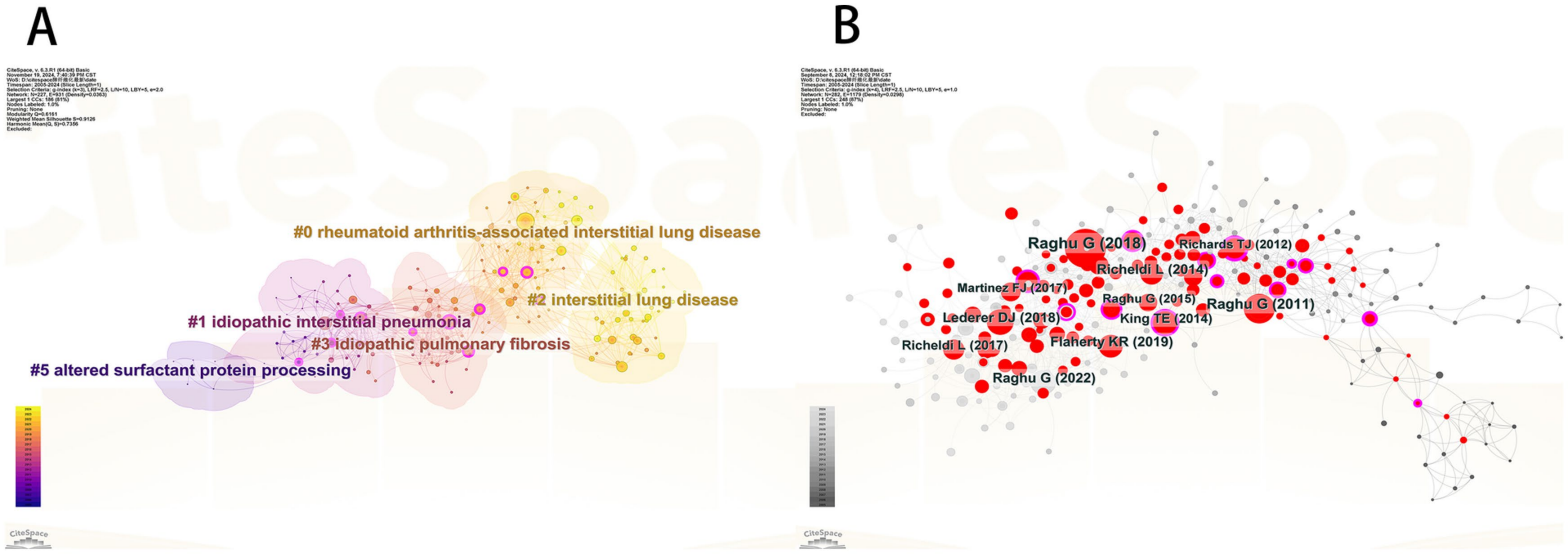
**(A)** Co-citation reference clustering diagram. **(B)** Literature co-citation analysis chart.

We compiled a Table 4 listing the top 10 most-cited articles and compared their Local Citations (TLCS) and Global Citations (TGCS). It is evident that the article “Raghu G, 2018, Am J Respir Crit Care” has the highest Local Citations (TLCS = 287). This article, a clinical practice guideline on the diagnosis of idiopathic pulmonary fibrosis published in the American Journal of Respiratory and Critical Care Medicine (19), reflects the high level of attention given by the U.S. academic community to diagnostic standards for pulmonary fibrosis. It also highlights that accurate diagnosis remains a major focus and challenge in current research. The article “Travis WD, 2013, Am J Respir Crit Care” has the highest Global Citations (TGCS = 2,810). This study systematically categorized idiopathic interstitial pneumonia and fibrosis, pointing out that molecular biomarker research is still needed to address the challenges in classifying some cases. It emphasizes the critical role of biomarkers in disease diagnosis, prognosis evaluation, and therapeutic research (20). We constructed a network relationship diagram (Figure 5A) to explore research directions. The diagram reveals five major clusters: rheumatoid arthritis-associated interstitial lung disease (#0), idiopathic interstitial pneumonia (#1), interstitial lung disease (#2), idiopathic pulmonary fibrosis (#3), and altered surfactant protein processing (#5). The co-occurrence of rheumatoid arthritis (#0) and interstitial lung diseases suggests that the interaction between autoimmune conditions and pulmonary fibrosis is a research hotspot. The high representation of idiopathic diseases (#1, #3) reflects the ongoing efforts to elucidate unknown etiologies. Cluster #5 highlights the integration of basic research (such as protein metabolism) with clinical phenotypes. An integrated analysis suggests that the mechanistic link between IPF (#3) and surfactant proteins (#5) could be a promising area for future breakthroughs.

**Table 4:** Citation frequency statistics of literature.

Number	Document	DOI	Year	Local citations	Global citations
1	RAGHU G, 2018, AM J RESP CRIT CARE	10.1164/rccm.201807-1255ST	2018	287	2,384
2	TRAVIS WD, 2013, AM J RESP CRIT CARE	10.1164/rccm.201308-1483ST	2013	232	2,810
3	ROSAS IO, 2008, PLOS MED	10.1371/journal.pmed.0050093	2008	177	423
4	RICHARDS TJ, 2012, AM J RESP CRIT CARE	10.1164/rccm.201101-0058OC	2012	122	286
5	PRASSEL A, 2009, AM J RESP CRIT CARE	10.1164/rccm.200808-1201OC	2009	111	254
6	MARTINEZ FJ, 2017, NAT REV DIS PRIMERS	10.1038/nrdp.2017.74	2017	107	787
7	MAHER TM, 2017, LANCET RESP MED	10.1016/S2213-2600(17)30430-7	2017	91	172
8	JENKINS RG, 2015, LANCET RESP MED	10.1016/S2213-2600(15)00048-X	2015	90	220
9	KINDER BW, 2009, CHEST	10.1378/chest.08-2209	2009	81	162
10	KONISHI K, 2009, AM J RESP CRIT CARE	10.1164/rccm.200810-1596OC	2009	78	258

To identify the most influential core literature in the field of pulmonary fibrosis research, we conducted a co-citation network analysis (Figure 5B). The analysis reveals that the article “Raghu G, 2018, Am J Respir Crit Care” has the largest node size, indicating that this paper has the broadest knowledge dissemination and academic impact within the field. As a clinical practice guideline published in the American Journal of Respiratory and Critical Care Medicine, its significant influence likely stems from providing an authoritative standardized framework for the diagnosis of pulmonary fibrosis, integrating the latest evidence-based medical data, and offering valuable guidance for both clinical practice and future research directions. This finding corroborates the central role of guideline-type literature in advancing the discipline, while also highlighting the importance of standardizing clinical diagnostic and treatment protocols in current pulmonary fibrosis research.

### Keyword analysis

**Figure 6:**
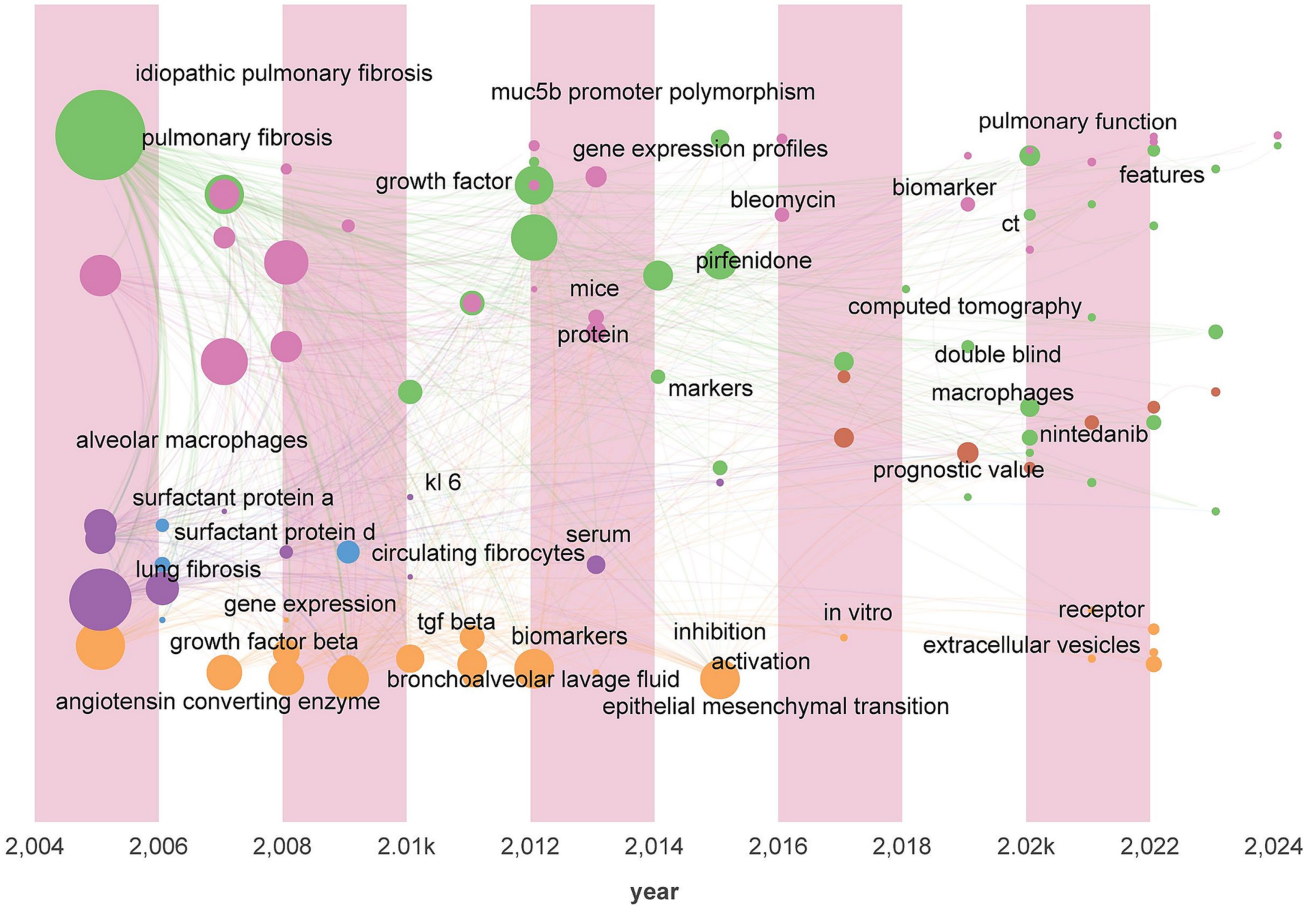
Keywords time zone chart.

Keyword analysis is an effective method for revealing the development trends in a research field. In this study we conducted temporal visualization analysis of the literature keywords using CiteSpace and Gephi software systematically illustrating the evolution of research on pulmonary fibrosis biomarkers. As shown in Figure 6 the analysis reveals the characteristics of the research development stages to date. Prior to 2018 the focus was primarily on exploring the basic pathophysiological mechanisms of pulmonary fibrosis and identifying biomarkers associated with the onset and progression of the disease such as alveolar macrophages SP-A/SP-D angiotensin-converting enzyme circulating fibroblasts gene expression MUC5B promoter polymorphisms and KL-6. However after 2018 new keywords such as CT nintedanib diagnostic value and double-blind appeared indicating a shift in research from basic mechanisms to the diagnostic and therapeutic domains with the term “double-blind” suggesting the initiation of relevant clinical studies. Furthermore we observed that the research on pulmonary fibrosis biomarkers has evolved from a singular to a multidimensional approach with gene biomarkers imaging biomarkers and protein biomarkers representing a multimodal fusion of omics. This indicates that researchers should consider the combined use of multiple biomarkers and the integration of various omics technologies when identifying and applying biomarkers.

**Figure 7:**
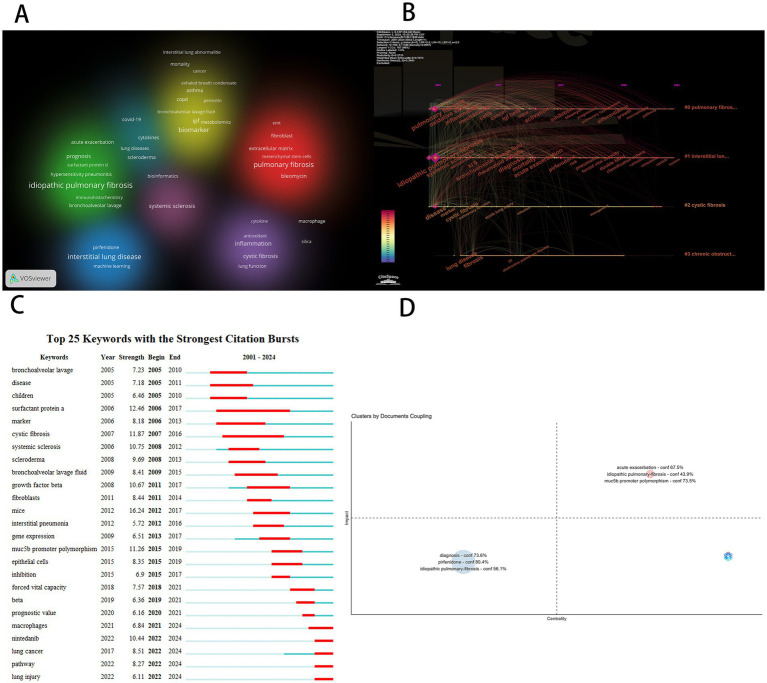
**(A)** Keyword clustering diagram. **(B)** Keywords timeline map. **(C)** Keyword prominence map. **(D)** Keyword coupling analysis diagram.

We conducted a cluster analysis of keywords based on their collaborative relationships and constructed a network relationship diagram (Figure 7A) to identify interdisciplinary or interactive research hotspots. As shown in the diagram, the keyword clustering groups “idiopathic pulmonary fibrosis” (IPF), “surfactant protein D” (SP-D), and “prognosis” into the same cluster, suggesting that SP-D, as a biomarker for IPF, may hold prognostic value. This finding provides a molecular-level direction for clinical disease progression monitoring. Additionally, pulmonary fibrosis and bleomycin are placed within the same cluster, indicating significant research focus on the relationship and mutual influence between these two elements. This clustering not only confirms known research trends (such as the clinical significance of SP-D) but also has the potential to inspire new research hypotheses. Future studies could explore these clustering relationships through more in-depth empirical analysis.

Subsequently, we conducted a temporal analysis of keywords using CiteSpace software and generated a keyword chronogram (Figure 7B) to reveal the evolutionary patterns of biomarkers across different research clusters. The results show that the keywords were grouped into four clusters: Pulmonary Fibrosis Research Cluster (#0): Core biomarkers include oxidative stress, mechanism, TGF-*β*, activation/inhibition, macrophages, and pathway. This cluster focuses on exploring the fundamental mechanisms of pulmonary fibrosis, reflecting that the pathogenesis of pulmonary fibrosis remains incompletely understood. Mechanistic research continues to be a key challenge and focus of current studies. Interstitial Lung Disease Research Cluster (#1): Key biomarkers include systemic sclerosis, rheumatoid arthritis, KL-6, acute exacerbation, risk, diagnosis, and nintedanib. This cluster highlights the importance of interstitial lung diseases as a significant subset of pulmonary fibrosis and underscores the challenges posed by unclear pathogenesis, high risk of acute exacerbation, and difficulties in diagnosis and treatment. Cystic fibrosis (#2): Keywords include acute lung injury, infection, and management. Cystic fibrosis is an autosomal recessive genetic disorder caused by mutations in the cystic fibrosis transmembrane conductance regulator (CFTR) gene, with a poor prognosis. Acute exacerbations caused by infections are a major cause of mortality. The keywords also emphasize the critical importance of long-term proper management for cystic fibrosis patients. Chronic Obstructive Pulmonary Disease (COPD) Cluster (#3): COPD and its comorbidity with pulmonary fibrosis represent an important clinical issue, significantly increasing mortality risk. Future research should focus on identifying biomarkers that can distinguish between COPD and pulmonary fibrosis comorbidity to enable earlier detection and intervention.

Keyword burst analysis is a powerful approach for identifying the evolution of research hotspots and emerging trends within a scientific domain. Using CiteSpace we performed a keyword burst analysis (Figure 7C) to systematically reveal the dynamic development of research on pulmonary fibrosis biomarkers. From a historical perspective the longest-lasting burst keyword was surfactant protein a (SP-A) which persisted from 2006 to 2017—A span of 11 years. SP-A a major component of pulmonary surfactant plays a crucial role in modulating alveolar surface tension as well as regulating immune and inflammatory responses it holds significant diagnostic and prognostic value in pulmonary fibrosis. SP-A levels in both serum and bronchoalveolar lavage fluid (BALF) have been shown to correlate with disease severity and can serve as predictors of patient survival. Moreover the combined use of SP-A with other biomarkers such as SP-D and KL-6 has been found to improve diagnostic accuracy and prognostic assessment (16). The temporal progression of burst keywords also reflects a shift in research paradigms. For instance the emergence of “marker” in 2006 indicates an early focus on biomarker identification. In 2013 the appearance of “gene expression” signaled a transition toward molecular-level investigations. By 2020 the emergence of “prognostic value” highlighted the expansion of biomarker applications into outcome prediction. Currently active burst keywords further illuminate the present research frontiers. For example macrophages play a crucial role in the initiation and progression of pulmonary fibrosis with changes in their polarization status and the secretion of various cytokines making them potential biomarkers (19). Previous studies have shown that during the early stages of fibrosis M1 macrophages primarily mediate inflammatory responses. However as the disease progresses macrophages transition to the M2 phenotype where they exacerbate the fibrosis process by secreting repair factors and promoting extracellular matrix deposition (20). Recent studies have begun to unravel the heterogeneous functions of macrophage subpopulations and their potential as therapeutic targets. Future research is expected to delve deeper into the molecular mechanisms and targeted therapies involving macrophages with the aim of improving clinical management. The keyword nintedanib marks the rise of anti-fibrotic drug development while lung injury and lung cancer point to growing interest in disease comorbidities particularly radiation induced pulmonary fibrosis and pneumonitis which are common complications in lung cancer patients undergoing radiotherapy and significantly affect treatment outcomes and prognosis (21). The keyword pathway underscores continued efforts to elucidate the molecular mechanisms underlying fibrosis. Collectively these burst patterns illustrate a clear developmental trajectory in the field: From early biomarker discovery to mechanistic elucidation drug development and clinical application with increasing emphasis on diagnostic biomarkers and therapeutic targets. This progression reflects both the maturation of the field and its ongoing transformation toward precision medicine.

Finally, we employed bibliometric coupling analysis and constructed a research topic network map (Figure 7D) using the Bibliometrix software to systematically assess the academic value of various research themes. The horizontal axis represents the centrality of a topic (reflecting its strength of association with other topics), while the vertical axis indicates the topic’s influence (demonstrating its contribution to the development of the field). The upper-right quadrant highlights core topics that exhibit both high centrality and high influence. As shown in Figure 7D, the research themes with the highest academic value are acute exacerbation and MUC5B promoter polymorphism. Notably, MUC5B promoter polymorphism, as a genetic biomarker, demonstrates prominent network positioning characteristics (centrality = 0.28, influence = 0.35), indicating its key role in the disease’s pathogenesis. It also shows strong associations with multiple other research themes, underscoring its significant clinical translational potential. Multiple genome-wide association studies have confirmed that the T allele mutation at the rs35705950 site in the promoter region of the MUC5B gene increases the risk of idiopathic pulmonary fibrosis (IPF) and shortens overall survival (22, 23). Moreover, its diagnostic sensitivity and specificity are significantly superior to those of traditional serum biomarkers. Longitudinal cohort studies further reveal that patients with MUC5B overexpression exhibit an increased annual decline in forced vital capacity (FVC) compared to the control group, with a significantly higher risk of progressing to end-stage lung disease (24, 25). Therefore, MUC5B is not only a biomarker for diagnosis and prognosis but also holds promise as a potential therapeutic target for pulmonary fibrosis (19, 26). Targeting MUC5B with new therapeutic strategies, such as neutralizing antibodies, may slow disease progression and improve patients’ quality of life. This analysis effectively identifies the core scientific questions in pulmonary fibrosis research and provides an objective basis for future research directions, particularly emphasizing the research value and clinical significance of genetic biomarkers in the acute exacerbation phase of pulmonary fibrosis.

## Discussion

### Challenges and opportunities of key biomarkers

As a potential biomarker for pulmonary fibrosis, MUC5B faces several challenges in the process of clinical translation. This is primarily due to its complex biological functions, which are regulated by genetic polymorphisms, resulting in significant interindividual differences (20, 27, 28). Currently, there is no standardized method for measuring MUC5B expression and function, and its molecular mechanisms remain incompletely understood (29, 30), which limits its practical application in clinical settings. However, advances in multi-omics technologies have provided new breakthroughs in MUC5B research (31–33). By integrating genomic, transcriptomic, and proteomic data, researchers are able to gain deeper insights into the characteristics of MUC5B and achieve more precise patient stratification. Combined with high-resolution CT imaging and radiomics, this approach allows for a more accurate reflection of the progression of pulmonary fibrosis, thus optimizing treatment strategies. Moreover, epigenomic studies have revealed the mechanisms through which MUC5B is regulated by environmental factors and lifestyle. The integration of epigenomic data with machine learning and imaging technologies holds promise for overcoming the challenges of MUC5B in clinical translation, offering significant support for early diagnosis and personalized treatment.

Macrophages play a critical role in the onset and progression of pulmonary fibrosis. They are not only involved in immune responses but also contribute significantly to tissue remodeling and fibrosis, thus serving a pivotal function in these processes (34–36). Furthermore, macrophages have the potential to be targeted for therapeutic intervention (37–39). However, the heterogeneity of macrophages presents a significant challenge to the development of targeted therapies. Different subpopulations of macrophages may exert distinct roles during the fibrosis process (40), thereby complicating the formulation of effective treatment strategies. To address this challenge, it is crucial to adopt a multi-dimensional research approach that integrates transcriptomics, epigenetics, single-cell RNA sequencing, proteomics, metabolomics, immunomics, and clinical data. A thorough analysis of these datasets could uncover the intricate mechanisms through which macrophages contribute to pulmonary fibrosis, thus providing new insights into precision-targeted therapies. For instance, single-cell RNA sequencing technology enables the precise identification of different macrophage subpopulations, allowing for a deeper investigation into their specific roles in the fibrotic process. Moreover, the application of network pharmacology provides a theoretical foundation for target discovery and drug optimization, driving the development of innovative therapeutic strategies.

### Current status and trend analysis of biomarkers in pulmonary fibrosis research

The research on biomarkers in pulmonary fibrosis is evolving toward a multidimensional integrated approach, moving beyond the confines of single diagnostic tools. Biomarker research has transitioned significantly from early serological markers (such as SP-A/SP-D) to the analysis of genetic features and imaging assessments (41–45). Most cases of pulmonary fibrosis progress from interstitial lung abnormalities (ILA) to chronic fibrotic interstitial lung disease (excluding acute lesions). The CT evolution of this process includes ground-glass opacities (GGO), reticular patterns, architectural distortion, traction bronchiectasis, and eventually honeycombing. CT not only guides biopsy and therapeutic decision-making but also predicts prognosis through fibrosis scoring and the traction bronchiectasis index (TBI) (43, 46). Radiomics and artificial intelligence can further quantify these lesions, although CT-based fibrosis quantification as an alternative endpoint still requires more research validation. In conclusion, despite the identification of various specific biomarkers (47–49), the heterogeneity in both the etiology and clinical presentation of pulmonary fibrosis still limits the universality and accuracy of existing biomarkers (50, 51). Therefore, future research should focus on the following key directions: (1) Multimodal Precision Medicine: By integrating genomic and imaging data, the development of prognostic biomarkers and dynamic monitoring systems for pulmonary fibrosis will be advanced, providing more personalized treatment options for patients. (2) Advances in Multi-Omics Technologies: Breakthroughs in genomics, transcriptomics, proteomics, and other fields will contribute to a deeper understanding of the etiology and pathogenesis of pulmonary fibrosis, providing theoretical support for the discovery of new biomarkers and therapeutic targets. (3) Exploration of Therapeutic Targets: Further investigation into the regulatory mechanisms of macrophages in fibrosis will be crucial to exploring novel therapeutic approaches, such as antifibrotic drugs and interventions targeting oxidative stress, and optimizing existing treatment strategies. (4) Clinical Translation and Application: Based on biomarker research, the updating of clinical guidelines and the acceleration of the translation of biomarkers from basic research to clinical practice will be essential to shorten the time from research findings to clinical application. The application of technologies such as genomics and artificial intelligence will provide new opportunities to overcome current diagnostic and therapeutic bottlenecks.

### Limitations of this study

This study uses the Web of Science Core Collection (WoSCC) as the data source, with indexing in SCI-EXPANDED and SSCI to ensure the accuracy of the retrieved data. However, this research relies solely on Web of Science as the literature search database, meaning that some literature from other databases (e.g., non-English databases) was not included in this study. Additionally, while we have made every effort to optimize the search terms, it is acknowledged that some relevant studies may have been overlooked. Finally, due to the timing of data analysis, literature published after August 2024 was not fully included, which resulted in a lack of analysis for studies published after this period. Nevertheless, this limitation does not impact the analysis of overall research topic trends.

## Conclusion

This study is the first to systematically conduct a bibliometric visualization analysis of research on pulmonary fibrosis biomarkers, highlighting the most representative countries, institutions, authors, journals, papers, and keywords in this field. It also organizes the research trends and hot topics. The analysis indicates that the evolution of research hotspots exhibits clear phases: before 2018, the focus was on basic mechanism exploration, while after 2018, there has been a shift toward clinical research and application. Further analysis reveals that genetic biomarkers and imaging-based biomarkers display unique characteristics at different stages of pulmonary fibrosis. Therefore, we can explore whether genetic and imaging biomarkers can become key elements in the comprehensive management of pulmonary fibrosis, particularly the clinical application value of radiomics integrated with genetic information. However, the field still faces several challenges: First, the clinical application rate of existing biomarkers is low, and many biomarkers lack validation in multi-center, large-sample studies. Second, there is no standardized protocol for the multimodal integration of radiomics and genetic biomarkers. Third, research on biomarkers for special subtypes, such as radioactive pulmonary fibrosis and interstitial pulmonary fibrosis, is lagging. Future efforts should focus on constructing international open databases, promoting interdisciplinary technological integration and international collaborations, and designing adaptive clinical trials to accelerate the translation of biomarkers. By deepening the “mechanism-diagnosis-therapy” research paradigm, the field of pulmonary fibrosis biomarkers is expected to achieve significant progress from disease stratification to personalized intervention.

## Data Availability

The original contributions presented in the study are included in the article/supplementary material, further inquiries can be directed to the corresponding authors.
